# Common complications and prevention strategies for resuscitative endovascular balloon occlusion of the aorta: A narrative review

**DOI:** 10.1097/MD.0000000000034748

**Published:** 2023-08-25

**Authors:** Yi Guan, Pinghao Chen, Hao Zhou, Jiaxiang Hong, Yanggang Yan, Yong Wang

**Affiliations:** a College of Pediatrics, Hainan Medical University, Haikou, China; b Department of Emergency, Hainan Clinical Research Center for Acute and Critical Diseases, the Second Affiliated Hospital of Hainan Medical University, Haikou, China; c Department of Interventional Radiology and Vascular Surgery, the Second Affiliated Hospital of Hainan Medical University, Haikou, China.

**Keywords:** complication, resuscitative endovascular balloon occlusion of the aorta, trauma

## Abstract

Resuscitative endovascular balloon occlusion of the aorta (REBOA) is considered a key measure of treatment due to its use in stabilizing patients in shock through temporary inflow occlusion for noncompressible torso hemorrhage as well as its supportive role in myocardial and cerebral perfusion. Although its clinical efficacy in trauma has been widely recognized, concerns over related complications, such as vascular access and ischemia-reperfusion, are on the rise. This paper aims to investigate complications associated with REBOA and identify current and emerging prevention or mitigation strategies through a literature review based on human or animal data. Common complications associated with REBOA include ischemia/reperfusion injuries, vessel injuries, venous thromboembolism, and worsening proximal bleeding. REBOA treatment outcomes can be improved substantially with the help of precise selection of patients, better visualization tools, improvement in balloon catheters, blockage strategies, and medication intervention measures. Better understanding of REBOA-related complications and further research on the strategies to mitigate the occurrence of such complications will be of vital importance for the optimization of the clinical outcomes in patients.

## 1. Introduction

About 1.5 million people die of trauma each year worldwide, with hemorrhagic shock being the major cause of death behind. Resuscitative endovascular balloon occlusion of the aorta (REBOA) blocks the flow of blood by placing a balloon in the aorta to control noncompressible torso hemorrhage (NCTH) through balloon dilation. In addition, REBOA can also improve the hemodynamics of the heart, brain and other important organs when used to control bleeding, which may extend the salvage window time and create favorable conditions for further definitive hemostasis operation or endovascular treatment.^[1,2]^ Although the efficacy of REBOA for trauma has been widely recognized compared to resuscitative thoracotomy^[3,4]^ or open aortic occlusion,^[5]^ the complications associated with REBOA emerge with a rising trend.^[6]^

In 2019, J. et al^[7]^ explored the database of the American College of Surgeons to carry out a case-control retrospective study for a comparison between trauma patients who received and did not receive REBOA, with 140 cases covered in the REBOA group while 280 cases in the non-REBOA group. According to the results, REBOA placement was associated with increased risks of mortality, lower extremity amputation, and acute renal injuries. Recently, a meta-analysis also showed that no significant difference was observed when REBOA was compared to no-REBOA.^[8]^ Therefore, concerted efforts are needed for a reassessment of the benefits and risks of REBOA. By now, no consensus has been reached concerning the complications arising from REBOA, as evidenced by different viewpoints and studies in this regard. To narrow the current gap in knowledge and improve outcomes related to REBOA, we investigated REBOA-induced complications and identified current and emerging prevention strategies through a literature review based on human or animal data.

## 2. Methods

For this review, we searched MEDLINE (PubMed), and EMBASE for articles published in English language between January 2010 and February 2023, using combinations of the terms “resuscitative endovascular balloon occlusion of the aorta,” “balloon occlusion,” “REBOA,” “hemorrhage,” “trauma,” “complication,” “prevention,” “mitigation.” All articles retrieved, including case reports, systematic review, clinical or animal studies, were further screened by abstract and/or full text, and relevant references cited in retrieved articles were also examined. Only those articles which investigated complication of REBOA and prevention or mitigation strategies of related complication were included. We also reviewed clinical practice guidelines for the implement of REBOA published by authorities, such as the American College of Emergency Physicians, the American College of Surgeons Committee on Trauma, and the Joint Trauma System and presented the possible resolution to minimize the risk of REBOA.

## 3. Results

### 3.1. REBOA clinical applications and practices

#### 3.1.1. Selection of patients.

REBOA is effective for NCTH below the diaphragm, including abdominal, retroperitoneal, pelvic, junctional or proximal lower extremity hemorrhage, especially for hemodynamic unstable patients.^[9,10]^ ROBOA should be avoided in those patients with head or thoracic injury,^[11,12]^ such as intracranial bleeding, thoracic vascular injury, pericardial tamponade, or hemothorax, because a few case reports showed that ROBOA may increase bleeding in sites proximal to occlusions, even in the case of minor injuries without active bleeding at the initial diagnosis.^[13,14]^. At present, some scholars have proposed to screen and select patients in need of REBOA treatment with the help of age, systolic pressure, Glasgow coma scale, and oxygen saturation. REBOA treatment brings significant clinical benefits under conditions where systolic pressure <90 mm Hg, Glasgow coma scale ≥9 points, or oxygen saturation >90%.^[15–17]^

#### 3.1.2. REBOA blockage zones.

The aorta involved can be divided into 3 anatomical Zones according to the blocking positions of REBOA. Zone I extends from the left subclavian artery to the part above the plane of peritoneal trunk, which can be used to control bleeding in abdominal and pelvic organs below the diaphragm, such as the bleeding induced by the rupture of liver, spleen, and kidney. Zone II extends from the peritoneal trunk to the plane of renal artery. As complex blood supply for abdominal organs is involved in this zone, it is usually not used as a balloon occlusion area. Zone III extends from the part below the plane of renal artery to the bifurcation of bilateral iliac arteries, which can be used to control bleeding in pelvic organs or induced by pelvic fracture or lower limb injuries.^[18]^

#### 3.1.3. REBOA operational steps.

In REBOA treatment, balloon catheters are placed into corresponding sections of the aorta to prevent the flow of blood to the distal end of the aorta after the balloons are dilated. In this manner, active bleeding beyond the blocked position is reduced while the afterload of the heart and the proximal aortic pressure are increased at the same time. This helps increase the perfusion in the heart and brain, thus winning more time to implement definitive hemostasis measures and further treatment.^[19]^ Specific operational steps are listed as follows: Establishment of the arterial access: A femoral arterial sheath is placed through percutaneous puncture or arterial cut-down. Placement of the balloon catheter: The balloon is inserted into the predetermined occlusion area guided by ultrasound or fluoroscopy. If no fluoroscopic or ultrasound devices are available at the site of first aid, REBOA was implemented using body surface landmarks, such as xiphoid or umbilicus to predict the length of REBOA catheter to be placed into the body.^[20]^ Inflation/deflation of the balloon: Physiological saline (or contrast agent) is injected into the balloon for inflation. The size and expansion pressure of the balloon are closely observed to avoid overinflation, and the proximal/distal arterial pressure is monitored. The balloon can be deflated at intervals. In addition, the balloon should be appropriately secured to avoid the migration of the balloon. Implementation of definitive resuscitative hemostasis: The position and duration of the blocked areas during the balloon occlusion are recorded. Removal of the balloon: The balloon is deflated before the balloon catheter is withdrawn. Removal of the femoral arterial sheath: The catheter sheath is removed, after which the puncture point is tended for hemostasis with the puncture site pressurized and bound for fixation.

### 3.2. Common REBOA-induced complications

The REBOA operation involves the establishment of the arterial access, the placement of the balloon, the dilation/fixing of the balloon, the management during balloon occlusion, the removal of the balloon, and the removal of the sheath tube, with possible occurrence of complications in each of the 6 steps.^[21]^

#### 3.2.1. Ischemia/reperfusion injuries of distal limbs and organs.

Most patients are suffering from hemorrhagic shock at the time of REBOA treatment, aortic inflow occlusion and recovery with the application of REBOA will further aggravate the ischemia/reperfusion injuries, including necrosis of distal organs (such as liver, kidney, intestines, and spinal cord) or limbs, loss of function, and even death.^[21]^ On top of that, ischemia/reperfusion injuries also lead to increased pressure in cavities, such as intra-abdominal hypertension,^[22]^ and compartment syndrome,^[23,24]^ both of which affect the final prognosis of the patients dramatically.

#### 3.2.2. Hematoma, pseudoaneurysm or arteriovenous fistula around the puncture site.

REBOA is usually performed via the femoral artery using Seldinger method or arteriotomy. Repeated punctures are usually required due to insufficient blood volume and collapsed arteries of the patient; Lager profile catheter and sheath are implanted in femoral artery; and most patients may suffer from coagulopathy during the ROBOA treatment. The aforesaid factors may induce complications as a result of the ROBOA puncture, among which hematoma, pseudoaneurysm, arteriovenous fistulas, and nerve injuries around the puncture site are more common. Femoral access complications include arterial disruption or dissection, thromboembolism and extremity ischemia were reported.^[25]^ A high puncture position may even lead to extremely dangerous retroperitoneal hematoma.^[21]^

#### 3.2.3. Vascular injuries.

Larger profile devices, inappropriate pushing of the guide wire and the guide catheter, incorrect placement or overinflation of the balloon, and an extremely long duration of balloon occlusion may lead to iatrogenic vascular injuries (vascular dissection, perforation, and rupture) along the operating path, thrombosis in vascular lumens, and embolism of distal organs and limbs.^[26,27]^ In addition to the rupture of the balloon under an over-dilated condition, the shear force generated at the instant of the rupture of the balloon also triggers aortic intimal injuries or dissection.^[21]^ Besides, since the sliding of the dilated balloon may also induce vascular injuries, appropriate management should be carried out after the placement of the balloon catheter.^[28]^

#### 3.2.4. Venous thromboembolism (VTE).

In severe trauma patients, coagulopathy may coexist with a hypercoagulable state. The tissue injury after hypoxia or ischemia provokes an inflammatory response, which comprises a cascade of endothelial injury, release of inflammatory cytokines, activation of platelet and leukocyte aggregation, and generation of thrombin and clot.^[29]^ Theoretically, occlusion of the arterial flow may result in venous stasis and increased VTE complications. W.Y.T. et al compared outcomes between 339 patients treated with REBOA and 663 patients with No REBOA, revealing that REBOA patients were significantly more likely to develop deep vein thrombosis and pulmonary embolism.^[30]^ C.C.Y. et al also found that REBOA in severe pelvic fractures had higher rates of in-hospital mortality, and higher rates of VTE.^[31]^

#### 3.2.5. Worsening proximal bleeding.

Rapid resuscitation in conjunction with aortic occlusion may result in an acute elevation in proximal blood pressures with worsening proximal bleeding. This may occur with REBOA implement and an unrecognized thoracic injury or cerebral injury.^[11,12]^

### 3.3. Prevention strategies for REBOA-induced complications

Note: In this chapter, most findings and results were based on animal experiments and preclinical studies. The regimen or devices mentioned in this chapter were not available clinically, and the benefit of those strategies needs to be investigated further and identified carefully.

#### 3.3.1. Intermittent or partial aortic balloon occlusion.

Currently, in clinical practices, further exploration is called for as regards how to achieve the balance between effective hemostatic effects and basic perfusion of blood at distal limbs and organs and whether partial or intermittent aortic balloon occlusion can extend the therapeutic window of REBOA, mitigate distal ischemia and thus improve the survival rate.^[32]^

Shortening the occlusion duration is the most important in reducing the treatment for ischemia arising from REBOA. As recommended by current guidelines, the total occlusion time for Zone I ranges between 30 and 45 minutes, with the occurrence of severe complications given a duration more than 60 minutes under most circumstances.^[33]^ Intermittent REBOA decreased the total ischemic time and increased the renal blood flow, urine output and decreased renal ischemic injury compared to continuous, complete aortic occlusion in a porcine model of uncontrolled class III hemorrhage.^[34]^ However, the balance between hemostasis and tissue perfusion cannot be achieved accurately in actual practices of intermittent REBOA.

Partial REBOA (pREBOA, also known as targeted regional optimization), allows certain blood flow through the balloon with targeted distal mean arterial pressure by controlling the size, shape and pressure of the balloon.^[35,36]^ This technology guarantees proximal arterial pressure and ensures distal blood perfusion, thus increasing the therapeutic window^[37]^ and reducing subsequent risk of traumatic exsanguination and distal ischemia.^[38]^ Significant mortality benefit with pREBOA was observed.^[39]^ According to relevant studies, complete REBOA increases afterload to facilitate resuscitation, but the penalty is supraphysiologic coronary flows and imposed increase in LV contractility to maintain cardiac output. pREBOA balances the increased afterload with improved aortic system compliance to prevent injury.^[40]^ In the meanwhile, as shown in an animal model of traumatic brain injury (TBI) and shock, pREBOA increased cerebral perfusion,^[41]^ but did not exacerbate TBI progression.^[42]^ However, pREBOA in combined TBI and shock may not be associated with better clinical outcomes.^[43]^ As shown in the cerebral infarction model, pREBOA can substantially increase the perfusion of blood in the ischemic area while decreasing the scope of cerebral infarction.^[44,45]^ In addition, recent evidence showed pREBOA had a significantly lower incidence of developing an AKI compared to routine REBOA in the United States.^[46]^

#### 3.3.2. Application of portable ultrasonic devices.

##### 3.3.2.1. Identification of indications.

If indications cannot be clearly identified through physical examination or computed tomography in the on-site first aid, portable ultrasonic systems can help determine whether there is bleeding and identify the source of bleeding. O.L.A. et al pointed out the effectiveness of ultrasound examination in locating the source of bleeding in thorax and ruling out contraindications, such as aortic injury, hematocele in thorax/mediastinum and pericardial tamponade.^[47]^

##### 3.3.2.2. Ultrasond-guided arterial puncture and balloon occlusion.

For patients suffering from hemorrhagic shock or cardiac arrest, it is apparently more difficult to acquire reliable percutaneous artery accesses. Therefore, establishment of arterial accesses with bedside ultrasound is recommended.^[48]^

Rapid and accurate placement of the balloon catheter during the REBOA treatment for resuscitation of patients suffering from massive bleeding after trauma is of great significance. REBOA is usually performed under the guidance of X-ray fluoroscopy. In case of pre-hospital setting without the aid of X-ray or critical conditions when it is dangerous to transport the patient, performing REBOA under the guidance of portable ultrasound will be faster, safer and more effective to identify the position of the balloon catheter, and observe the flow in the artery before and after balloon inflation.^[49,50]^.

##### 3.3.2.3. Evaluation of vascular access complications.

The use of ultrasound examination for vascular access before and after the removal of the catheter sheath can detect pseudoaneurysm and other complications in an early stage, and curb the occurrence of arterial or vein thrombosis to a large extent.^[47]^

#### 3.3.3. Development of new balloons.

At present, the commonly used balloon catheter is 7 to 14 Fr in diameter. Balloon catheters with low profile can improve the safety of REBOA while reducing the occurrence of vascular access injuries and embolism at distal limbs.^[51]^ G.S. et al proposed that short balloons are able to prevent the occurrence of viscera ischemia; compared with balloons with a standard size, the beneficial effects of short balloons on the heart and coronary arteries still remain when they are used for balloon occlusion in aorta.^[52]^ A prototype balloon system was a fused wire and balloon catheter scheme. The lead or insertion end is a floppy tipped 0.035-inch wire fused to the compliant balloon catheter. A collapsible nitenol rail system is positioned between the floppy tip of the wire and the compliant balloon for the purpose of centering the system within the axial arterial lumen as the device is inserted and positioned without the aid of fluoroscopy guidance.^[53]^ As pREBOA appears to be a promising technique, new pREBOA devices with proprietary configuration of multiple balloons or lobes were specifically designed to allow for fine and titratable control of aortic flow across the spectrum from balloon inflation to deflation.^[54–56]^

#### 3.3.4. Slow release of balloon pressure.

In the case of rapid release of the balloon catheter, since the sudden loss of aortic afterload has caused the redistribution of circulating blood, a large amount of blood will reenter the distal blood vessels that are highly dilated due to severe ischemia. Consequently, a large number of ischemic metabolites, such as interleukin 6,^[57]^ will return to rejoin the systemic circulation, which may lead to inflammation-mediated organ failure. Therefore, it is recommended to release the dilated balloon slowly in a continuously controllable and gradual manner.^[32]^

#### 3.3.5. Drug intervention.

Tissue injuries and organ dysfunction can be reduced with the help of sulfide pretreatment, which weakens inflammation as well as oxidative and nitrosation stress reactions.^[57]^ S.F. et al demonstrated that erythropoietin helps improve renal function and spinal cord integrity during the ischemia-reperfusion induced by aortic occlusion.^[58]^ T.S. et al discovered that urinary trypsin inhibitor improves renal oxygenation after reperfusion without altering the cardiopulmonary parameters.^[59]^ F.F.A. et al revealed that the infusion of physiological saline deteriorates the gas exchange caused by lung reperfusion injuries.^[60]^ Sevoflurane, a narcotic drug, eases hemodynamic sequelae caused by reperfusion injuries arising from balloon catheter occlusion in thoracic aorta with better effects compared to propofol and reduces the release of serum markers for cell injuries in the meanwhile.^[61]^

#### 3.3.6. Best practice of access intervention.

The clinician may access the common femoral artery with a blind percutaneous approach or surgical cut-down. If a percutaneous approach is preferred, it is strongly recommended to perform with ultrasound guidance. To prevent falling out eventually, the sheath and catheter must be secured in multiple sites and covered with an occlusive dressing. When REBOA is no longer required, the deflated balloon should be removed from the arterial sheath. Prior to sheath removal, the sheath should be flushed with heparinized saline. At the time of sheath removal, direct pressure should be held over the arterial entry site instead of the skin puncture site. Vascular closure instrument is recommended for those sheaths larger than 7 French. Open approach requires open surgical vascular repair.

## 4. Conclusion

To sum up, as an effective auxiliary hemostatic technology, REBOA not only improves cerebral and myocardial perfusion and promotes resuscitation but is also conducive to emergency treatment of traumatic NCTH. Many trauma centers in the United States have adopted REBOA as a temporary measure of treatment for patients suffering from subdiaphragmatic NCTH.^[58]^

Common complications associated with REBOA include ischemia/reperfusion injuries at distal limbs and organs, hematoma, pseudoaneurysm or arteriovenous fistulas around the puncture site, arterial injuries along the passway (dissection and thrombosis), and venous thromboembolism etc. Bedside ultrasound guidance, new types of balloons and occlusion schemes, and drug interventions will ultimately bring more clinical benefits to patients by continuously lowering the risks of ROBOA.

## Author contributions

**Conceptualization:** Yanggang Yan, Yong Wang.

**Data curation:** Yi Guan, Pinghao Chen, Hao Zhou, Jiaxiang Hong.

**Formal analysis:** Yi Guan, Pinghao Chen, Hao Zhou, Jiaxiang Hong.

**Funding acquisition:** Yong Wang.

**Investigation:** Yi Guan, Pinghao Chen, Hao Zhou, Jiaxiang Hong, Yong Wang.

**Methodology:** Yi Guan, Pinghao Chen.

**Supervision:** Yanggang Yan, Yong Wang.

**Writing – original draft:** Yi Guan, Pinghao Chen, Hao Zhou.

**Writing – review & editing:** Yanggang Yan, Yong Wang.
